# Are Doctors so Very Bad?

**Published:** 1891-01-31

**Authors:** 


### The Hospital

A Weekly Journal of

Science, Medicine, Nursing, and Philanthropy.

*For Hospitals, Asylums, Infirmaries, Dispensaries, Medical Practitioners, Students, Nurses, and the Charitable Public.*

Vol. IX.—No. 227.] [January 31, 1891.

### Are Doctors so Very Bad?

"Tell Dr. B. not to send any more Hospitals. It is a bigoted paper, which holds the belief that unless a man is diplomaed he can't know anything. I am a free trader in physic as in everything, and the day must soon come when none but wealthy people will see doctors at their own houses for continued treatment." This candidly expressed opinion upon the medical profession in general and ourselves in particular is sent to us by a North country physician. It is not, we hasten to say, the opinion of the physician himself, but that of a near relative, who is following the profession of a civil engineer in the West Indies. Some of our readers will probably be considerably startled, as we were, to find that The Hospital is regarded by certain non-medical readers as an extreme exponent of high and dry professional orthodoxy. But we are disposed to let that pass. There seems to be no particular reason at the present moment for defining our own position. We are quite willing that readers who wish to have it defined shall define it for themselves. It is the latter part of the civil engineer's opinion which is of general importance; and his decidedly expressed conviction that a man without a medical diploma may be quite as good a practitioner as a man with one, and perhaps a great deal better, is worth refutation.

The opinion that an unqualified medical practitioner has a certain kind of presumptive evidence in his favour in the fact that he holds his own, and sometimes makes more money than the legally qualified man, is an opinion which concerns the general public much more than it does the members of the medical profession. Speaking as a doctor, the writer neither hates nor fears unqualified practitioners. He feels that if he, with his education and experience, and of course his degree, cannot do better than the man who does not possess those advantages, there must be a fault somewhere. He certainly ought to do better, and on the whole he has a decided conviction that he can. At any rate, he is willing to take his chance in the open struggle of life against the great unqualified. This we believe to be the position of the vast majority of the members of the medical profession. They are willing to stand or fall by their own character and work.

But, for some reason, there is evidently a prejudice in the minds of certain honest and excellent persons against those who are known as orthodox practitioners. The fact that the editor of *The Review of Reviews* should so conspicuously espouse the cause of Count Mattei, and should affirm that "it is discreditable to the intelligence of the profession, and the credit of men who have the life and death of patients in their hands, to refuse to subject claims so influentially supported, on evidence so indisputable, to verification"—this fact is of some significance, and demands the consideration of those who have a just regard for the public fame and reputation of medicine. But, we repeat, that much as these questions may concern the thirty or forty thousand doctors who practise in the British dominions, they concern the five times thirty or forty millions of the general population a great deal more.

Is it a right and proper thing for a boy to learn his trade? And is he likely to learn it all the better if he knows that he will be subjected to certain tests of competency before he can be allowed to practise it on his own account? And when he has learnt it and has passed his tests, is it to be expected that another boy or man who has neither passed the tests nor been apprenticed to the business will know as much about it as he does? The thing is too absurd. Medical men are not a race by themselves; if they were, it might be allowable to argue that they are greater fools than other people; and, therefore, that the common axioms of "every man to his trade," "the tools to him who can handle them," and the like, do not apply to doctors; although everybody admits that it is proper to apply them to all other professions and callings whatsoever. But doctors are not a race by themselves; they are chosen from among the general population; and, therefore, it is most natural to suppose that they have the same average of brains and conscience as the general population. To suppose they have less is absurd; to suppose they have more is not necessary for our argument. What would our civil engineer say if, when he was building a bridge, a self-confident doctor should step up to him, and, telling him to "stand aside," should proceed to carry on the work on his own account? It is to be expected and hoped, for the sake of his manhood, that the engineer would be somewhat less "civil" than his designation might lead us to expect.

All that is very well, say the supporters of the unqualified: but the "quacks" succeed in curing, where the "orthodox" fail. Which is just as true and wise as it would be to say that the commerce of the world had better be carried on in small open boats because one American happened to cross the Atlantic in such a boat, than in iron steamers of five thousand tons. The general claim that quacks cure where the orthodox do not, reaches the very pinnacle and maximum of absurdity. The reason, of course, why a foolish and unreflecting public should have come, even for a moment, to think such a thing true, is that the quacks proclaim, or invent their cures, whilst the orthodox do not. The quack in fact lives by his proclamations; the orthodox lives by his honest daily work and his self respect. Mr. W. T. Stead, the editor of *The Review of Reviews*, seems to think that he has made out a striking case against orthodox doctors and in favour of Count Mattei; and he is surprised that the doctors do not take up and investigate the Count's claims without a moment's delay. We, on our part, are quite as much surprised that, with all his zeal and skill, he has made out such an exceedingly poor case for the Count. For let us consider one or two plain facts. The Count has been curing cancer, so it is said, for thirty years: well then he should by this time have a large number of cases of such a kind to report as that there can be no manner of doubt about either their quantity or their quality. It would not be too much to ask, for example, that, since his remedy is so certain and efficacious, and his patients are, and have long been, so numerous in every country, he should have cured at least one case a week: that is to say, fifty-two a year; or altogether, fifteen hundred and sixty cases in thirty years. This we say would not be an unreasonable demand to make. But what are the actual facts? Mr. Stead has visited Count Mattei at his home; and has interrogated him at full length upon all the doings of his life: and has seen Dr. Kennedy who appears to be the only medical man in this country who has put the Count's remedies to a test. What is the winnowed result of Mr. Stead's investigations? This: that he records less than a single dozen of well attested cures of cancer, and gives us a few extracts from letters of eminent persons, some of whom, perhaps, had received, and more were willing to believe that they had received, benefit from the Count's treatment.

Now, if we could get at the records of any eminent British surgeon who has been in the full practice of his profession for thirty years we would engage to show that, though he had often failed to restore cancerous patients to health, yet for every single case of *bonâ fide* cancer that has been cured or relieved by Count Mattei's treatment during that time, at least twenty cases have been cured or relieved by our eminent British surgeon: twenty for one, and more even than that. Of the truth of this statement we have no more doubt than we have of the statement that people have skated on the upper reaches of the Thames during the late severe frost.

The truth is that the public do not in the least know the honesty, devotion to duty, and the high aims of the worthiest medical practitioners of the present time. Neither Thomas à Kempis, nor any other saint, ever devoted himself to the highest objects of his religion with more loyalty and fidelity than do some of the British physicians and surgeons of our own times to the intense and mind-exhausting labours of curing disease and averting death. We, too, are free traders; we forbid no man; we warn no intruder off the special preserves of medicine; let everyone who can cure do so by all means. But the unqualified practitioner in general, and Count Mattei in particular, are no more to be compared with the average orthodox practitioner and the eminent physicians and surgeons of our own period, for worth of character and practical efficiency, than is the unwashed chimney-sweep to be compared for whiteness of skin with one of the baby princes in a Royal nursery. Cant is always and everywhere hateful; and never more hateful than when it stands by the bedside of the sick and the dying and pretends to recall them to life with lies and knavery. Whoso believes that we have a professional axe to grind is welcome to his credulity.

